# Label Noise Cleaning with an Adaptive Ensemble Method Based on Noise Detection Metric

**DOI:** 10.3390/s20236718

**Published:** 2020-11-24

**Authors:** Wei Feng, Yinghui Quan, Gabriel Dauphin

**Affiliations:** 1 Department of Remote Sensing Science and Technology, School of Electronic Engineering, Xidian University, Xi’an 710071, China; wfeng@xidian.edu.cn; 2 Laboratory of Information Processing and Transmission, L2TI, Institut Galilée, University Paris XIII, 93430 Paris, France; gabriel.dauphin@univ-paris13.fr

**Keywords:** classification, label noise, supervised learning, ensemble learning, multiclass

## Abstract

Real-world datasets are often contaminated with label noise; labeling is not a clear-cut process and reliable methods tend to be expensive or time-consuming. Depending on the learning technique used, such label noise is potentially harmful, requiring an increased size of the training set, making the trained model more complex and more prone to overfitting and yielding less accurate prediction. This work proposes a cleaning technique called the ensemble method based on the noise detection metric (ENDM). From the corrupted training set, an ensemble classifier is first learned and used to derive four metrics assessing the likelihood for a sample to be mislabeled. For each metric, three thresholds are set to maximize the classifying performance on a corrupted validation dataset when using three different ensemble classifiers, namely *Bagging, AdaBoost* and *k*-nearest neighbor (*k*-NN). These thresholds are used to identify and then either remove or correct the corrupted samples. The effectiveness of the ENDM is demonstrated in performing the classification of 15 public datasets. A comparative analysis is conducted concerning the homogeneous-ensembles-based majority vote method and consensus vote method, two popular ensemble-based label noise filters.

## 1. Introduction

In machine learning, the prediction accuracy depends not only on the appropriate choice of the learning technique, but also on the quality of the database. Quoting [1], “real-world databases are estimated to contain around five percent of encoding errors, all fields taken together when no specific measures are taken.” Noise-contaminating databases can be mainly of two types: feature noise or label noise, also called class noise (i.e., mislabeled data) [2]. Whether one or the other prevails depends on the application field. Using inaccurate sensors or choosing less invasive measurements may explain why the feature noise is predominant. On the other hand, labeling training instances may be contaminated with data entry errors. It is a costly and rather subjective task as the meaning of a label could be inadequate [3,4,5,6,7,8,9,10], As a result, the label noise could be predominant.

Feature noise is generally spread over many features and each feature noise component tends to be statistically independent of the others, and most learned classifiers are robust to such noise. Conversely, label noise can significantly affect the learning performance [11,12,13] and should be taken into account when designing learning algorithms [14]. Noise can increase the number of necessary training instances, the complexity of learned models, the number of nodes in decision trees [1] and the size (number of base classifiers) of an ensemble classifier [5]. Learning from noisy labeled data can also create overfitting [15]. Incorrectly labeled examples may severely bias the learning method and result in inaccurate models [5].

As learning from noisy labeled data is a challenging issue [3], an important subfield of the literature is devoted to its study [1,16]. It follows mainly three ideas: finding robust learning techniques [17,18], postprocessing data by adapting learning techniques, and preprocessing data by removing some instances, or sometimes by correcting some labels. Many Bagging-based [19] ensemble techniques are more robust learning techniques as compared to the support vector machine (SVM) [20], which uses support vectors, AdaBoost [21], which happens to give more weight to mislabeled samples, or *k*-nearest neighbor (*k*-NN) [22], especially when only one neighbor is considered (k=1).

This first idea—“finding robust learning techniques”—is generally regarded as less effective [3]. The second idea—“postprocessing data by adapting learning techniques”—is an active research topic that includes probabilistic methods derived from the mislabeling random assumption and model-based methods, attempting to avoid the consequences of label noise [1]. The third idea—“removing or correcting some instances”, which we follow in this work—was already popular in the eighties [17]. It consists of designing filters, detecting mislabeled instances and removing them [4,23] or correcting them [24,25]. Often with some similarities to outlier detection, such noise filters focus on the instances that are difficult to classify and are easy to use [26]. When filtering a training set, two conflicting difficulties are encountered:

1. (**Case 1**) A clean sample is regarded as mislabeled and cleaned. This case harms the classification performance, especially when the size of the training dataset is small.
2. (**Case 2**) A mislabeled sample is regarded as clean and retained or unchanged. This makes noisy samples remain in the training dataset and degrades the classification performance.

The ensemble approach is a popular method to filter out mislabeled instances [4,15,23,27,28,29]. It constructs a set of base-level classifiers and then uses their classifications to identify mislabeled instances [11]. The majority filter and consensus filter are two typical noise cleaning methods. A majority filter tags an instance as mislabeled if more than half of the base classifiers do not predict the right label. A consensus filter tags an instance as mislabeled if all base classifiers do not predict the right label. When using the consensus filter whose criterion is strict, only a small portion of the label noise is removed. Most mislabeled instances then remain in the filtered training set and performance is hindered, more than when using the majority vote filter, as it removes a higher portion of the label noise. Because of the diversity of the ensemble classifier used in these majority filter, samples near the classification boundary have a reduced amount of base classifiers predicting the right label, and more correctly, labeled instances are removed from the training set, which can negatively affect the classifier’s performance [1,30].

Depending on whether base classifiers are induced using different or similar learning techniques, the ensemble-based noise filtering method is referred to as heterogeneous or homogeneous.

In the heterogeneous method, an ensemble classifier detects mislabeled instances by constructing a set of base-level detectors (classifiers) and then using their classification errors to identify mislabeled instances. Brodley et al. chose three well-known algorithms from the machine learning and statistical pattern recognition communities to form the filters: decision trees, nearest neighbor classifiers, and linear machines. An instance is tagged as mislabeled if α of the *T* base-level classifiers cannot classify it correctly. In heterogeneous ensembles, the decision borders are varied because the individual classifiers are of different types. The dispersion of class noise may reflect this variability. Hence, this method tends to eliminate instances that lie on the wrong side of the classification boundary, which can negatively affect the classifier’s performance.

The homogeneous ensemble vote for noise filtering [15] is an improved version of the aforementioned heterogeneous ensemble-based method. Verbaeten and Assche considered the problem of mislabeled training examples by preprocessing the training set based on some well-known ensemble classification methods (Bagging and boosting) [15] using C4.5 as base classifier [31]. They proposed two approaches:

1. Filtering based on voting (consensus vote and majority vote) of base classifiers of a Bagging ensemble.
2. Filtering based on removing training examples that obtained high weights in the boosting process. Indeed, mislabeled examples are assumed to have high weights.

Results show that majority vote filters are still more accurate than consensus filters in the homogeneous ensemble method. In addition, Bagging majority vote filters outperform the boosting filters. The boosting filter tends to incorrectly remove many important correctly labeled instances with large weights. In addition, in a homogeneous ensemble, the decision boundaries of the individual classifiers are similar to each other. Then, noisy examples close to this decision boundary can be detected effectively by the majority voting [32]. In summary, in this paper, the homogeneous ensemble-vote-based method for noise filtering is used as the comparison in the experiment.

As an example of the ensemble approach but in a different manner, there is outlier removal boosting (ORBoost) [33], where data cleaning is performed while learning, and not after nor before. The only difference with AdaBoost is that the weight assigned to each sample is set to zero when it exceeds a certain threshold. Good performance is observed when the label noise is low.

An ensemble classifier induced on a training set is also a precious information source on each instance: how many base classifiers did not predict correctly? What is the cumulative weight of these base classifiers failing to predict the assigned label? To what extent might the ensemble classifier have been able to predict a different label? These questions have greatly influenced our work. Using a technical definition of an edge [34,35] exploited the answers to the two first questions to detect mislabeled instances. [23] exploited the answer to the third question to compute for each instance a margin with which mislabeled instances are detected. However, an important amount of mislabeled instances are not removed, perhaps because the possibly correct label of instances from the training set have actually no impact on the margin value and the amount of suspicious instances removed is set upon the performance on a validation dataset using Bagging, which happens to be fairly robust to label noise. Our work is different in that (1) to set the amount of removed samples, instead of only Bagging, it adopts three methods (Bagging, AdaBoost and *k*-NN, with k=1 rendering it especially noise-sensitive); (2) four noise detection metrics are considered (instead of one); (3) the ENDM is extended to label noise correction. A comparative analysis is also conducted concerning the majority vote filter [4,15].

In the paper, we deal with the label noise issue using an adaptive ensemble method based on a noise detection metric (ENDM). It is called adaptive because there is no fixed threshold being used to select the suspicious instances as for majority and consensus filters—rather, there is a counting parameter whose value is selected using a validation set. Our proposed method for noise detection is described in Section 2. In Section 3, we present the results of an empirical evaluation of the proposed method and a comparison with other state-of-the-art noise detection methods. In Section 4, the conclusions are provided, and future works are discussed.

## 2. Label Noise Cleaning with an Adaptive Ensemble Method Based on Noise Detection Metrics

### 2.1. Label Noise Detection Metric

**Figure 1:**
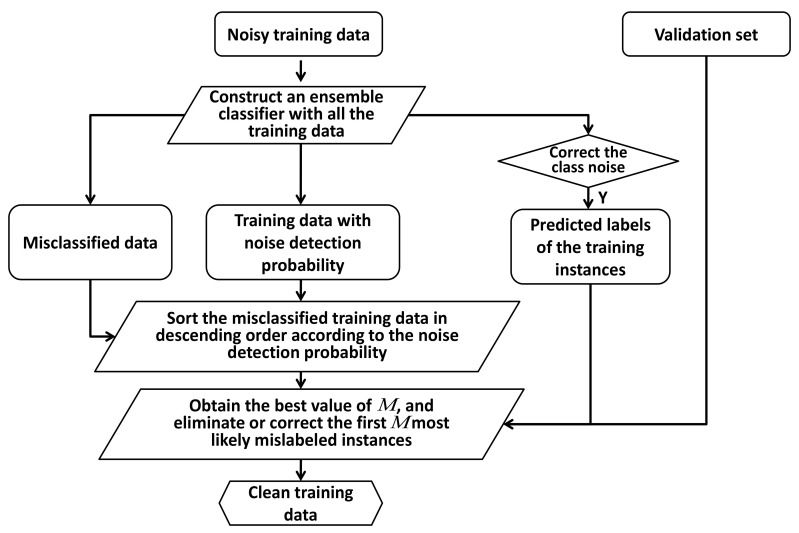
Flowchart of label noise cleaning with an adaptive ensemble method based on a noise detection metric.

In this work, it is assumed that all instances have an unknown, yet fixed, probability of being misclassified. This assumption is called the uniform label noise in [1]. To assess whether an instance is more likely to be misclassified than another, a homogeneous ensemble classifier is trained and four metrics are computed based on the votes of the base classifiers and their number [5]. Once samples are ordered according to one of the metrics, it suffices to use a validation set to fix the exact number of tagged instances and hence to select the suspicious instances [23]. The flowchart of the proposed method is shown on Figure 1.

Let us define some notations,

- ζ is an ensemble model composed of *T* base classifiers,
- (x,y) is an instance, with x as a feature vector and *y* as one of the *C* class labels,
- $S=\left\{\left(x_{1},y_{1}\right),\cdots ,\left(x_{n},y_{N}\right)\right\}$ is a set of *N* training samples,
- v(x,c) is the number of classifiers predicting the label *c* when the feature vector is x,
- Λ(x,y) is a metric assessing the likelihood that a sample (x,y) will be mislabeled (it is used to sort samples),
- 1(P) is equal to one when statement P is true and is equal to zero otherwise,
- λ counting parameter.

All four metrics proposed are ranging from 0 to 1, 0 indicating that the label is very suspicious and 1 indicating that this label is reliable.

#### 2.1.1. Supervised Max Operation (SuMax)

A popular ensemble margin function was introduced by Schapire et al. [36] and has been used in data importance evaluation [5]. This ensemble margin is defined as

(1) $${\mathrm{margin}}_{\mathrm{SuMax}}\left(x,y\right)=\frac{1}{T}\left(v\left(x,y\right)-\max_{c\neq y}v\left(x,c\right)\right)$$

A positive value of ${\mathrm{margin}}_{\mathrm{SuMax}}\left(x,y\right)$ means that this instance is correctly classified by the set of *T* classifiers when using a majority vote. A negative value indicates misclassification. When there is a class *c* such that v(x,c) equals to *T*, margin (x,y)=1 or margin (x,y)=−1 depending on whether c=y or not. Otherwise, the range of margin(x,y) is (−1,1).

The SuMax-based noise detection metric is defined by Equation (2).

(2) $$\Lambda_{\mathrm{SuMax}}\left(x,y\right)=\left|{\mathrm{margin}}_{\mathrm{SuMax}}\left(x,y\right)\right|=\frac{1}{T}\left|v\left(x,y\right)-\max_{c\neq y}v\left(x,c\right)\right|$$

#### 2.1.2. Supervised Sum Operation (SuSum)

The margin of a sample is also obtained by the difference between the fraction of classifiers voting correctly and incorrectly [37]. Unlike the previous definition, in a multiclass context, a negative value of the margin does not necessarily indicate misclassification. This definition bears some resemblance with the definition of an edge in [34]: Given an ensemble classifier and an instance, the edge is the *sum* of the weights associated to classifiers predicting a wrong label.

The SuSum-based noise detection metric is defined by Equation (3).

(3) $$\Lambda_{\mathrm{SuSum}}\left(x,y\right)=\left|{\mathrm{margin}}_{\mathrm{SuSum}}\left(x,y\right)\right|=\frac{1}{T}\left|v\left(x,y\right)-\sum_{c\neq y}v\left(x,c\right)\right|$$

#### 2.1.3. Unsupervised Max Operation (UnMax)

In [23], the authors proposed a new margin definition that is more robust to label noise. It is an unsupervised version of Schapire’s method and defined in Equation (4).

(4) $$\Lambda_{\mathrm{UnMax}}\left(x\right)={\mathrm{margin}}_{\mathrm{UnMax}}\left(x\right)=\frac{1}{T}\left(v\left(x,\zeta \left(x\right)\right)-\max_{c\neq \zeta (x)}v\left(x,c\right)\right)$$

where ζ(x) is the predicted class of the ensemble classifier for sample x: $\zeta \left(x\right)={arg max}_{c}v\left(x,c\right)$.

#### 2.1.4. Unsupervised Sum Operation (UnSum)

In our previous work [38], we proposed a new unsupervised data importance evaluation method. It is an unsupervised version of the SuSum (3) and is defined in Equation (5).

(5) $$\Lambda_{\mathrm{UnSum}}\left(x\right)=\left|{\mathrm{margin}}_{\mathrm{UnSum}}\left(x\right)\right|=\frac{1}{T}\left|v\left(x,\zeta \left(x\right)\right)-\sum_{c\neq \zeta (x)}v\left(x,c\right)\right|$$

According to the above presentations of the four metrics, the supervised margins need the true label of the mislabeled instances while the unsupervised margins are robust to the true class values. In addition, when compared with the max operation, the sum based methods tend to give the misclassified instances with higher noise weight values.

### 2.2. Label Noise Cleaning Method

The proposed label noise cleaning method can be used with any of the four noise detection metrics. The samples tagged as mislabeled are the λN samples having the smallest metric values, with λ itself depending on whether the tagged samples are removed or corrected.

#### 2.2.1. Label Noise Removal with *ENDM*

The pseudo-code of the *ENDM* based noise removal method is presented as Algorithm 1.

In the first step, *Bagging* is used to induce an ensemble classifier composed of pruned trees from the whole training set. Collect in $S^{\prime }$ the misclassified samples. Based on predictions of base classifiers, the chosen metric is used to sort samples in $S^{\prime }$ according to their metric value. Compute $\lambda_{\max}$ defined as the ratio of the size of $S^{\prime }$ to the size of *S*.

The second step is an iterative procedure. In this step, λ ranges from 0 to $\lambda_{\max}$ in steps of 1%. At each iteration, a new subset containing the correctly classified samples of *S* and the λN-lowest sorted samples. Then, this obtained subset is used to learn a classifier (*Bagging*, *AdaBoost* or *k-NN* with k=1), and the accuracy of this classifier is measured on the validation set. The finally selected λ-value is the one yielding the highest accuracy of the validation set.

In the last step, a clean training set is defined with the selected λ-value as in the second step, and a noise-sensitive classifier, *AdaBoost* or *k-NN*, is, induced on this clean training set, and tested on the test set.
**Algorithm 1** Label-noise removal with an adaptive ensemble method based on noise-detection metric1:**Input:**2:Training set $S=\{\left(x_{1},y_{1}\right),\left(x_{2},y_{2},\right),\cdots ,\left(x_{N},y_{N}\right)\}$;3:Validation set *V*;4:Noise-robust ensemble classifier algorithm A;5:Noise-sensitive ensemble classifier algorithm B;6:Choose a metric, $\Lambda \in \{\Lambda_{\mathrm{SuMax}},\Lambda_{\mathrm{UnMax}},\Lambda_{\mathrm{SuSum}},\Lambda_{\mathrm{UnSum}}\}$;7:**Process:**8:Train ζ according to A with *S*;9:Collect in set $S^{\prime }$ all misclassified instances, $S^{\prime }:=\left\{\left(x,y\right)\in S|\zeta \left(x\right)\neq y\right\}$;10:Compute the maximum value of λ, $\lambda_{\max}:=\frac{|S^{\prime }|}{\left|S\right|}$;11:Sort instances in $S^{\prime }$ according to Λ in decreasing order;12:**for**λ=0:$\lambda_{\max}$**do**13: Initiate clean set with the correctly labeled instances, $S_{\lambda }:=S\setminus S^{\prime }$;14: Fill in $S_{\lambda }$ the λN-lowest samples, $S_{\lambda }:=S_{\lambda }⋃\left\{\left(x_{i},y_{i}\right)|i\leq \lambda N\right\}$;15: Train $\zeta_{\lambda }$ according to B with $S_{\lambda }$;16: Compute accuracy of $\zeta_{\lambda }$ on *V*, $a_{\lambda }:=\frac{1}{|S_{\lambda }|}\sum_{\left(x,y\right)\in S_{\lambda }}1\left(y=\zeta_{\lambda }\left(x\right)\right)$17:**end for**18:Select the optimal λ-value: $\hat{\lambda }=arg maxa_{\lambda }$;19:Select the best filtered training set: $S^{\prime \prime }=S_{\hat{\lambda }}$.20:**Output:**21:The clean training set $S^{\prime \prime }$.

#### 2.2.2. Label Noise Correction with *ENDM*

The only difference with *ENDM*-based noise correction is in that tagged samples have their label corrected instead of being removed. Its pseudo-code is presented as Algorithm 2 and its description is also divided into three steps.

In the first step, the same technique is used to induce an ensemble classifier (*Bagging* with pruned trees), and the same misclassified samples are collected in $S^{\prime }$ and sorted according to the metric chosen. The value $\lambda_{\max}$ is the ratio which is used to control the number of the removed instances.

In the second step, λ ranges from 0 to $\lambda_{\max}$ by step of 1%. Then, the first λN-highest sorted samples of $S^{\prime }$ are tagged. This step is different in that instead of collecting the nontagged samples in a new subset, it is the whole training set that is considered and the labels of the $S^{\prime }$ nontagged samples are changed into those predicted by the ensemble classifier. This modified training set is again used to learn a classifier (*Bagging*, *AdaBoost* or *k-NN* with k=1), and the accuracy of this classifier is measured on the validation set. As the inducing sets have been modified, there is no reason that the selected λ-value yielding the highest accuracy should be the same.

The last step is the same, though the inducing set is different in size and label values: a noise-sensitive classifier, *AdaBoost* or *k-NN*, is induced on this modified training set and tested on the test set.
**Algorithm 2** Label noise correction with an adaptive ensemble method based on noise-detection metric1:**Input:**2:Training set $S=\{\left(x_{1},y_{1}\right),\left(x_{2},y_{2},\right),\cdots ,\left(x_{N},y_{N}\right)\}$;3:Validation set *V*;4:Noise-robust ensemble classifier algorithm A;5:Noise-sensitive ensemble classifier algorithm B;6:Choose a metric, $\Lambda \in \{\Lambda_{\mathrm{SuMax}},\Lambda_{\mathrm{UnMax}},\Lambda_{\mathrm{SuSum}},\Lambda_{\mathrm{UnSum}}\}$;7:**Process:**8:Train ζ according to A with *S*;9:Collect in set $S^{\prime }$ all misclassified instances, $S^{\prime }:=\left\{\left(x,y\right)\in S|\zeta \left(x\right)\neq y\right\}$;10:Compute the maximum value of λ, $\lambda_{\max}:=\frac{|S^{\prime }|}{\left|S\right|}$;11:Sort instances in $S^{\prime }$ according to Λ in decreasing order;12:**for**λ=0:$\lambda_{\max}$**do**13: Initiate clean set with *S*, $S_{\lambda }:=S$;14: Modify in $S_{\lambda }$ with A the λN-highest samples, $\forall i\leq \lambda N,y_{i}:=A\left(x_{i}\right)$;15: Train $\zeta_{\lambda }$ according to B with $S_{\lambda }$;16: Compute accuracy of $\zeta_{\lambda }$ on *V*, $a_{\lambda }:=\frac{1}{|S_{\lambda }|}\sum_{\left(x,y\right)\in S_{\lambda }}1\left(y=\zeta_{\lambda }\left(x\right)\right)$17:**end for**18:Select the optimal λ-value, $\hat{\lambda }:=arg maxa_{\lambda }$;19:Select the best filtered training set, $S^{\prime \prime }:=S_{\hat{\lambda }}$.20:**Output:**21:The clean training set $S^{\prime \prime }$.

## 3. Experimental Results

### 3.1. Experiment Settings

As in [2,4,5,15,27] and actually following most of the literature addressing the noise label issue, artificial noise is introduced in the training set and the validation set, not in the test set. In all our experiments, 20% of the training samples and 20% of the validation samples are randomly selected and have their labels randomly modified to another label. For a fairer comparison, we included the validation data in the training data when the validation set was not necessary (e.g., no filtering, majority vote filters and consensus vote filters). As for the *Bagging*-induced ensemble classifier using the uncleaned training set, in all experiments, it is composed of exactly 200 pruned Classification and Regression Trees (*CART*) [39] as base classifiers.

**Figure 2:**
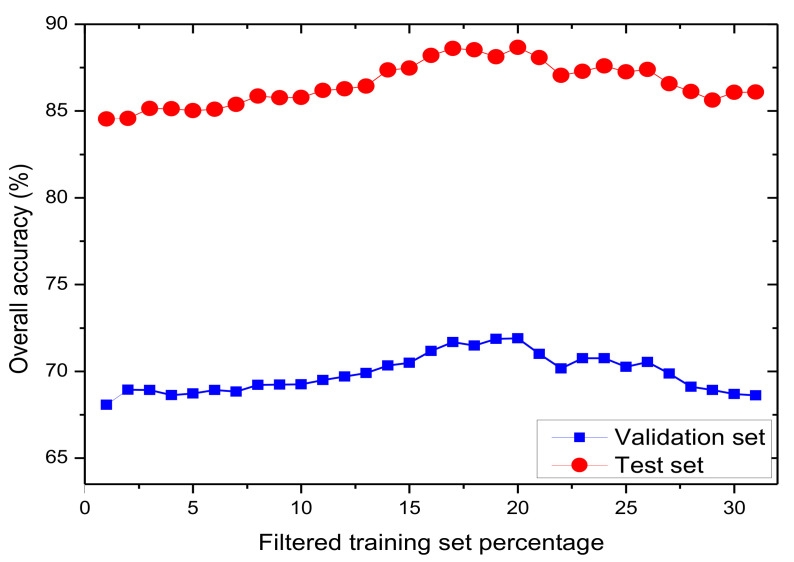
Classification accuracy of *AdaBoost* on dataset *Statlog*’s validation set (20% randomly selected of its labels are randomly modified) and test set (no labels are modified) as a function of λ (ratio of tagged labels).

To exemplify the proposed method, it is applied with noise removal on Statlog dataset with λ ranging from 0 up to $\lambda_{\max}\left(=31\%\right)$ by step of 1%. Subsets are built by collecting the λN-lowest values of the training set. *AdaBoost* is induced on each of those subsets. Figure 2 shows two curves. The lower is the accuracy measured on the validation set, of each induced *AdaBoost* classifier, as a function of λ. The upper is the accuracy measured on the test set of the same classifiers as a function of λ. As the test set is noise-free, it is no surprise that the test-set measured accuracy is significantly higher than the validation-set measured accuracy. Note that both curves have very similar shapes, showing the appropriateness of the proposed way of selecting λ.

A comparative analysis is conducted between the *ENDM*-based mislabeled data identification method and the homogeneous-ensemble-based majority vote method [15]. Both label noise removal and correction schemes are involved in the comparison.

### 3.2. Datasets

The experimentation is based on 15 public multiclass datasets from the UCI and KEEL machine learning repository [40]. Each dataset was divided into three parts: training set, validation set and test set. Those datasets are described in Table 1, where Num. refers to the number of examples, Variables to the number of attributes (and their type) and Classes to the number of classes.

### 3.3. Comparison of *ENDM* Versus no Filtering

Table 2 and Table 3 show respectively the accuracy of *AdaBoost* and *k-NN*-classifiers induced using three different training sets: the training set is not modified (no filtering), the training set is *ENDM*-based filtered with noise removal and noise correction. On all 15 datasets using both learning techniques and regardless of the modality chosen (noise removal or noise correction), the *ENDM* technique is better performing than not using any filter with an average increase of 2.42%. In comparison with not filtering, the *ENDM*-technique yields an increase in accuracy of 10% on dataset *Letter* with *AdaBoost* and about 16% with *k-NN* on dataset *Optdigit*. The *ENDM* noise-correction modality, be it with *AdaBoost* or *k-NN*, appears to be most often less performing than the noise removal modality; nonetheless, it remains better than not using any filter.

### 3.4. Comparison of *ENDM* Versus Other Ensemble-Vote-Based Noise Filter

Table 2 and Table 3 also show the accuracy of *AdaBoost* and *k-NN* classifiers, respectively, induced using different training sets: the training sets are majority-vote-based and consensus vote-based filtered with noise removal and noise correction, and the training set is *ENDM*-based filtered with noise removal and noise correction. As noted previously, the noise-correction modality of the majority-vote-based noise filtering technique is most often less performing than the noise-removal modality when using *AdaBoost* or *k-NN*. On 10 of the 15 datasets, using *AdaBoost*, and considering only the noise-removal modality, *ENDM* is more successful than the other ensemble-vote-based noise filtering techniques. On dataset *Letter*, the increase is of 9% using *AdaBoost* and 25% using *k-NN*. Both tables show that *ENDM* is more safe with respect to majority vote method. The majority-vote-based and consensus-vote-based methods tend to tag more instances as noisy. When the data removal is carried out, more useful samples are wasted. With respect to the majority vote filter, the best increase in accuracy is, respectively, over **9%** and **25%** with *AdaBoost* on dataset *Letter*.

**Figure 3:**
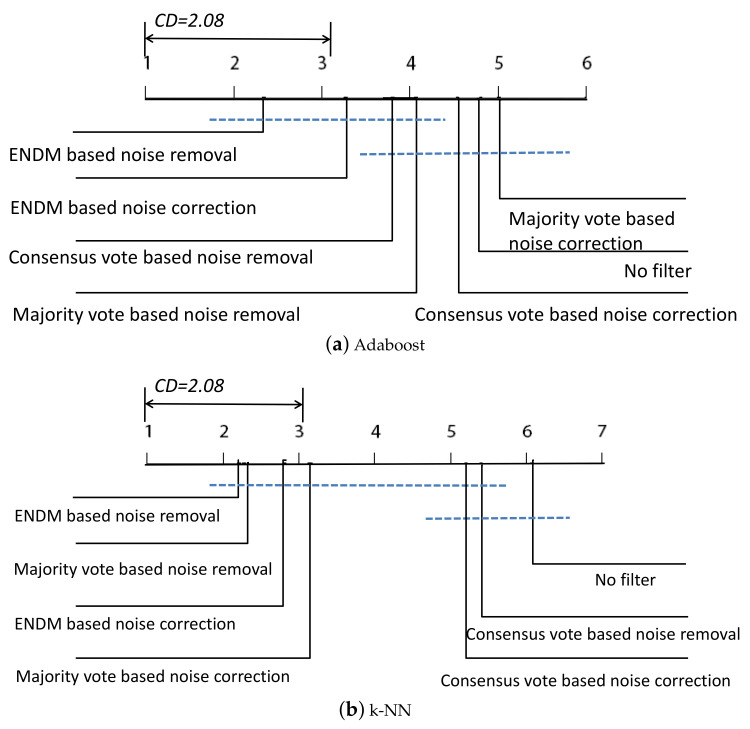
Bonferroni-Dunn (95% confidence level) for the comparative methods on all data sets.

To further analyze the performance of the proposed method, a nonparametric statistical test, the Friedman test, Refs. [41,42] is used. Table 4 and Table 5 have provided a summary of the mean ranks of all algorithms. To verify whether the proposed method performs better than the reference algorithms, the critical difference (CD) is adopted by the Bonferroni-Dunn post-hoc test. Figure 3 presents the results of post-hoc tests for comparative algorithms over all the multiclass datasets. According to the results of the Bonferroni Test, the proposed method presents a good performance in dealing with the class noise problem of the multiclass datasets. Furthermore, noise removal outperforms noise correction for all ensemble-based methods.

### 3.5. Comparing Different Noise Detection Metrics in *ENDM*

**Figure 4:**
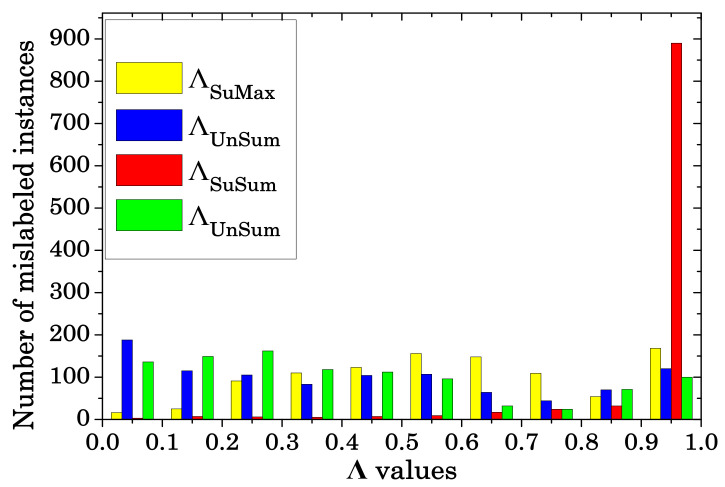
Evaluation of the Overall Accuracy *Letter* according to the ensemble size, *T*.

**Figure 5:**
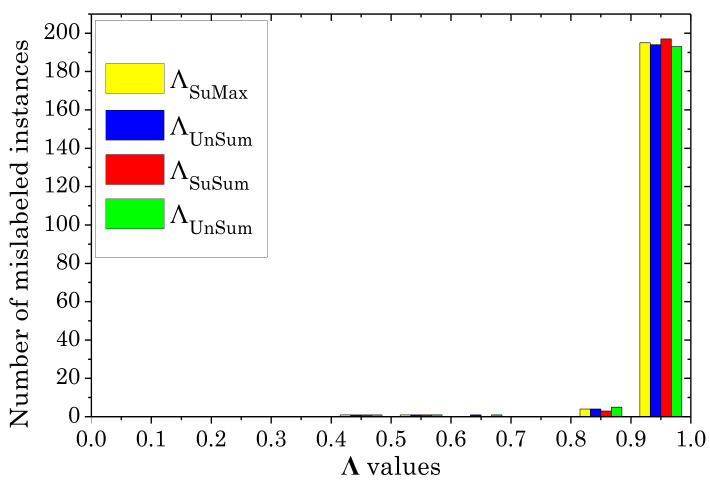
Evaluation of the Overall Accuracy on *Optdigits* according to the ensemble size, *T*.

Table 6 and Table 7 also show the accuracy of *AdaBoost* and *k-NN*-classifiers, respectively, induced using twelve different *ENDM*-based filtered training sets with the noise-removal modality: three different classifiers (*Bagging*, *AdaBoost*, *k-NN* with k=1) are used to select λ and four different metrics (SuMax, UnMax, SuSum, UnSum) yield four different ways of ordering samples. The histogram figures of the performances of the four different noise detection metrics in the proposed algorithm on the datasets *Letter* and *Optdigits* are shown in Figure 4 and Figure 5.

When comparing columns 1 and 2 or 3 and 4 from both tables, it appears that using supervised metrics (SuMax and SuSum) are most often more successful than using unsupervised metrics (UnMax and UnSum). When comparing columns 3 and 1 from both tables, it appears that using SuSum is more successful than SuMax quite often. This finding does not extend to the unsupervised metrics, as UnSum and UnMax yield similar performances. This may be explained by the lack of information available to unsupervised metrics.

### 3.6. Comparing Different Classifiers Used for λ-Selection in *ENDM*

Table 6 and Table 7 show the accuracy of AdaBoost and *k*-NN classifiers, respectively, induced using twelve different ENDM-based filtered training sets with the noise removal modality: four different metrics (SuMax, UnMax, SuSum, UnSum) and three different classifiers (Bagging, AdaBoost and *k*-NN with k=1) are used to select λ. Table 8 and Table 9 show the accuracy of AdaBoost and *k*-NN, respectively, when both classifiers are combined with the proposed noise correction modality.

When comparing the accuracies yielded using the Bagging-selected λ-value with those yielded using AdaBoost and *k*-NN, it appears that Bagging is most often not the appropriate tool to select λ. For example, in Table 7, when compared with bagging, the AdaBoost/*k*-NN could increase the accuracy of over 11% on the Vehicle. Actually, Bagging is known to be more label-noise-robust than AdaBoost and *k*-NN when k=1, and λ is estimated as the value yielding the best performance when trained on a λ-dependent training set and tested on a validation set. Therefore, it makes sense to use noise-sensitive classifiers instead of noise-robust classifiers.

In Table 6, when comparing the AdaBoost-tested accuracies yielded using the AdaBoost-selected λ-value with those yielded using the *k*-NN-selected λ-value, AdaBoost seems more appropriate to select λ. Now, in Table 7, when comparing the *k*-NN-tested accuracies yielded using the AdaBoost-selected λ-value with those yielded using the *k*-NN-selected λ-value, *k*-NN seems more appropriate to select λ. Hence, when the classifier used on the clean training set is noise-sensitive, it seems sensible to use that same learning technique when selecting λ.

## 4. Conclusions

This paper has focused on cleaning training sets contaminated with label noise, a challenging issue in machine learning, especially when a noise-sensitive classifier is desired. In line with the literature, we have proposed a two-stage process called ENDM, where an induced ensemble classifier enables the measuring of label’s reliability of each training instance, and then the maximization of the accuracy of a second classifier tested on a validation provides an estimate of the number of samples that should be removed or corrected. When compared on 15 public datasets, with no cleaning, with the majority-vote-based filtering method, and with the consensus-vote-based filtering method, ENDM appears to perform significantly better.

In addition, experiments and discussion have provided some insights into how a label’s reliability should be measured, whether suspicious samples should be removed or have their labels modified and how the second classifier ought to be chosen.

Future work will investigate more realistic ways of introducing artificial label noise in the datasets. Imbalanced label-noisy datasets will also be considered as cleaning filters tend to be more discriminative against minority instances, being more difficult to classify.

## Figures and Tables

**Table 1:** Data sets.

Data Set	Training Set Num.	Validation Set Num.	Test Set Num.	Variables	Classes
Abalone	1500	750	1500	8	3
ForestTypes	200	100	200	27	4
Glass	80	40	80	10	6
Hayes-roth	64	32	64	3	3
Letter	5000	2500	5000	16	26
Optdigits	1000	500	1000	64	10
Penbased	440	220	440	15	10
Pendigit	2000	1000	2000	16	10
Segment	800	400	800	19	7
Statlog	2000	1000	2000	36	6
Texture	2000	1000	2000	40	11
Vehicle	200	100	200	18	4
Waveform	2000	1000	2000	21	3
Wine	71	35	72	12	3
Winequalityred	600	300	600	11	6

**Table 2:** Accuracy of the *AdaBoost*-classifier induced using 15 different training sets: three filtering techniques (no filter, majority-vote and *ENDM*) and two modalities (noise removal and noise correction). The best results are marked in bold.

Data	No Filter	Label Noise Removal			Label Noise Correction		
		Majority Vote	Consensus Vote	ENDM	Majority Vote	Consensus Vote	ENDM
Abalone	53.85	53.92	54.27	**54.64**	54.19	54.36	54.61
ForestTypes	84.50	84.40	84.70	**84.83**	84.50	83.40	83.20
Glass	98.00	97.50	**98.25**	97.92	97.50	97.75	96.50
Hayes-roth	65.00	64.38	64.06	59.38	64.06	**65.31**	60.94
Letter	46.72	47.83	47.96	**56.88**	45.59	45.22	50.88
Optdigits	89.32	90.84	88.98	**94.14**	87.98	88.78	93.23
Penbased	90.05	**93.05**	90.36	91.89	92.45	90.00	92.23
Pendigit	90.34	92.95	91.73	**95.40**	89.56	90.80	93.64
Segment	92.12	91.13	93.73	**94.90**	91.11	93.47	94.41
Statlog	83.38	85.68	86.27	**88.75**	83.66	86.00	88.15
Texture	86.31	89.52	88.11	**94.03**	85.83	87.43	91.42
Vehicle	72.20	73.70	73.20	73.00	**74.30**	71.80	73.80
Waveform	81.44	79.09	81.53	**82.97**	77.07	81.70	82.41
Wine	90.83	92.78	89.17	91.67	93.33	91.11	**93.89**
Winequalityred	60.90	60.67	60.70	60.83	60.60	**61.47**	60.30
Average	79.00	79.83	79.53	**81.42**	78.78	79.24	80.64

**Table 3:** Accuracy of *k-NN*-classifier induced using 15 different training sets: three filtering techniques (no filter, majority-vote and *ENDM*) and two modalities (noise removal and noise correction). The best results are marked in bold.

Data	No Filter	Label Noise Removal			Label Noise Correction		
		Majority Vote	Consensus Vote	ENDM	Majority Vote	Consensus Vote	ENDM
Abalone	45.07	**53.53**	49.27	51.53	53.40	49.47	52.33
ForestTypes	65.00	78.00	65.00	77.50	**80.00**	65.00	76.00
Glass	63.75	73.75	63.75	70.00	**76.25**	63.75	72.50
Hayes-roth	45.31	53.12	37.50	46.88	**57.81**	40.62	51.56
Letter	74.62	59.08	75.02	**85.12**	63.20	74.72	80.44
Optdigits	77.90	**93.30**	79.20	93.10	88.70	79.80	90.40
Penbased	80.23	93.41	80.45	**94.77**	92.50	80.91	93.18
Pendigit	79.75	95.05	85.45	**96.30**	89.80	85.80	94.15
Segment	81.54	90.30	86.50	**93.30**	89.12	86.38	93.02
Statlog	73.35	84.95	83.10	**87.15**	81.40	83.20	85.80
Texture	80.21	94.42	87.10	**95.51**	87.32	86.90	93.24
Vehicle	59.00	66.50	59.00	**71.50**	66.50	59.00	67.50
Waveform	62.75	**78.40**	64.80	77.45	75.20	65.55	75.70
Wine	79.17	**94.44**	79.17	88.89	93.06	79.17	88.89
Winequalityred	49.67	58.67	48.33	57.33	**61.00**	48.00	58.17
Average	67.82	77.79	69.58	79.09	77.02	69.88	78.19

**Table 4:** Accuracy rank of *AdaBoost*-classifier on 15 different training sets using no filter, majority-vote, consensus-vote and *ENDM*) for both noise removal and noise correction.

Data	No Filter	Label Noise Removal			Label Noise Correction		
		Majority Vote	Consensus Vote	ENDM	Majority Vote	Consensus Vote	ENDM
Abalone	7	6	4	1	5	3	2
ForestTypes	3	5	2	1	3	6	7
Glass	2	5	1	3	5	4	7
Hayes-roth	2	3	4	7	4	1	6
Letter	5	4	3	1	6	7	2
Optdigits	4	3	5	1	7	6	2
Penbased	6	1	5	4	2	7	3
Pendigit	6	3	4	1	7	5	2
Segment	5	6	3	1	7	4	2
Statlog	7	5	3	1	6	4	2
Texture	6	3	4	1	7	5	2
Vehicle	6	3	4	5	1	7	2
Waveform	5	6	4	1	7	3	2
Wine	6	3	7	4	2	5	1
Winequalityred	2	5	4	3	6	1	7
Average rank	4.80	4.07	3.80	2.33	5.00	4.53	3.27

**Table 5:** Accuracy rank of *k-NN*-classifier on 15 different training sets using no filter, majority-vote, consensus-vote and *ENDM*) for both noise removal and noise correction.

Data	No Filter	Label Noise Removal			Label Noise Correction		
		Majority Vote	Consensus Vote	ENDM	Majority Vote	Consensus Vote	ENDM
Abalone	7	1	6	4	2	5	3
ForestTypes	5	2	5	3	1	5	4
Glass	5	2	5	4	1	5	3
Hayes-roth	5	2	7	4	1	6	3
Letter	5	7	3	1	6	4	2
Optdigits	7	1	6	2	4	5	3
Penbased	7	2	6	1	4	5	3
Pendigit	7	2	6	1	4	5	3
Segment	7	3	5	1	4	6	2
Statlog	7	3	5	1	6	4	2
Texture	7	2	5	1	4	6	3
Vehicle	5	3	5	1	3	5	2
Waveform	7	1	6	2	4	5	3
Wine	5	1	5	3	2	5	3
Winequalityred	5	2	6	4	1	7	3
Average rank	6.07	2.27	5.40	2.20	3.13	5.20	2.80

**Table 6:** Accuracy of the *AdaBoost* classifier trained on *ENDM*-based filtered training set with the noise-removal modality, four different metrics (SuMax, UnMax, SuSum, UnSum) and three different classifiers applied on the validation dataset (*Bagging*, *AdaBoost*, *k-NN* with k=1). The values in brackets are the detected noise ratios. The best results are marked in bold.

		SuMax	UnMax	SuSum	UnSum
Letter	*Bagging*	48.13 (12%)	52.83 (30%)	**53.08 (24%)**	49.60 (28%)
	*AdaBoost*	52.04 (22%)	49.60 (24%)	**56.88 (30%)**	50.54 (26%)
	*k-NN* (k=1)	**51.72 (21%)**	46.79 (16%)	51.03 (16%)	49.35 (7%)
Optdigits	*Bagging*	93.00 (17%)	90.71 (12%)	90.65 (10%)	**93.57 (18%)**
	*AdaBoost*	93.43 (15%)	93.15 (22%)	**94.14 (20%)**	93.23 (20%)
	*k-NN* (k=1)	**93.45 (22%)**	92.13 (23%)	93.10 (21%)	91.97 (25%)
Pendigit	*Bagging*	91.23 (6%)	91.25 (5%)	**94.98 (21%)**	91.41 (7%)
	*AdaBoost*	95.27 (20%)	94.24 (23%)	**95.40 (18%)**	93.87 (25%)
	*k-NN* (k=1)	**94.90 (20%)**	93.90 (25%)	94.70 (22%)	92.93 (22%)
Statlog	*Bagging*	86.50 (10%)	86.44 (9%)	**87.97 (22%)**	86.46 (10%)
	*AdaBoost*	**88.75 (22%)**	86.68 (13%)	88.66 (20%)	86.98 (14%)
	*k-NN* (k=1)	**88.66 (22%)**	86.90 (29%)	87.65 (26%)	85.98 (30%)
Vehicle	*Bagging*	72.10 (14%)	**72.90 (6%)**	70.35 (8%)	72.60 (7%)
	*AdaBoost*	72.30 (20%)	72.05 (17%)	72.05 (18%)	**73.00 (13%)**
	*k-NN* (k=1)	**72.80 (6%)**	72.25 (10%)	70.85 (8%)	72.15 (20%)

**Table 7:** Accuracy of the *k-NN* (k=1) classifier trained on *ENDM*-based filtered training set with the noise-removal modality and four different metrics (SuMax, UnMax, SuSum, UnSum) and three different classifiers (*Bagging*, *AdaBoost*, *k-NN* with k=1) applied on the validation dataset. The values in brackets are the detected noise ratios. The best results are marked in bold.

		SuMax	UnMax	SuSum	UnSum
Letter	*Bagging*	79.32 (14%)	78.22 (18%)	**80.88 (30%)**	76.64 (6%)
	*AdaBoost*	77.68 (35%)	73.52 (35%)	**79.00 (35%)**	76.52 (5%)
	*k-NN*	79.94 (23%)	78.18 (13%)	**85.12 (16%)**	77.60 (8%)
Optdigits	*Bagging*	90.60 (17%)	85.30 (10%)	**91.90 (20%)**	87.30(14%)
	*AdaBoost*	90.30 (18%)	88.40 (15%)	**92.70 (24%)**	89.10 (19%)
	*k-NN*	93.00 (25%)	92.90 (25%)	**93.10 (23%)**	93.00 (25%)
Pendigit	*Bagging*	87.40 (7%)	89.15 (9%)	**96.05 (23%)**	87.40(7%)
	*AdaBoost*	95.90 (21%)	95.80 (22%)	**96.25 (20%)**	95.20 (24%)
	*k-NN*	**96.30 (22%)**	95.90 (25%)	95.85 (19%)	94.20 (20%)
Statlog	*Bagging*	84.20 (13%)	81.00 (9%)	**86.55 (22%)**	83.60 (12%)
	*AdaBoost*	**86.80 (23%)**	84.90 (15%)	85.95 (18%)	85.90 (23%)
	*k-NN*	87.10 (22%)	86.40 (25%)	**87.15 (23%)**	86.85 (29%)
Vehicle	*Bagging*	59.50 (2%)	**60.00 (2%)**	59.00 (2%)	59.00 (2%)
	*AdaBoost*	70.00 (14%)	65.50 (12%)	**71.50 (22%)**	67.50 (14%)
	*k-NN*	63.00 (4%)	64.00 (9%)	**70.00 (17%)**	66.00 (13%)

**Table 8:** Accuracy of the *AdaBoost* classifier trained on the *ENDM*-based filtered training set with the noise-correction modality, four different metrics (SuMax, UnMax, SuSum, UnSum) and three different classifiers (*Bagging*, *AdaBoost*, and *k-NN* with k=1). The values in brackets are the detected noise ratio. The best results are marked in bold.

		SuMax	UnMax	SuSum	UnSum
Letter	*Bagging*	**50.94 (1%)**	50.85 (1%)	46.02 (0%)	50.88(1%)
	*AdaBoost*	**50.44 (1%)**	**50.44 (1%)**	48.40 (0%)	50.32 (1%)
	*k-NN*	43.56 (9%)	**46.11 (7%)**	41.81 (15%)	44.00 (8%)
Optdigits	*Bagging*	89.80 (0%)	**90.10 (0%)**	89.87 (0%)	89.80 (0%)
	*AdaBoost*	**93.23 (16%)**	92.30 (15%)	92.21 (18%)	91.33 (17%)
	*k-NN*	92.36 (17%)	**92.73 (16%)**	92.17 (19%)	90.51 (22%)
Pendigit	*Bagging*	90.89 (6%)	90.76 (3%)	90.04 (0%)	**91.05 (6%)**
	*AdaBoost*	93.57 (19%)	91.84 (16%)	**93.64 (18%)**	91.57 (12%)
	*k-NN*	92.11 (15%)	91.00 (14%)	**93.08 (18%)**	91.11 (14%)
Statlog	*Bagging*	86.48 (14%)	86.02 (16%)	84.83 (0%)	**86.50 (12%)**
	*AdaBoost*	87.80 (20%)	86.39 (9%)	**88.15 (19%)**	86.38 (13%)
	*k-NN*	86.58 (18%)	86.16 (16%)	**86.92 (23%)**	86.24 (16%)
Vehicle	*Bagging*	**73.90 (1%)**	73.75 (1%)	73.50 (0%)	72.95 (0%)
	*AdaBoost*	**73.85 (17%)**	72.00 (20%)	73.55 (17%)	72.80 (23%)
	*k-NN*	**73.85 (11%)**	72.90 (2%)	73.05 (8%)	72.95 (2%)

**Table 9:** Accuracy of the *k-NN* classifier trained on the *ENDM*-based filtered training set with the noise correction modality, four different metrics (SuMax, UnMax, SuSum, UnSum) and three different classifiers (*Bagging*, *AdaBoost*, and *k-NN* with k=1). The values in brackets are the detected noise ratio. The best results are marked in bold.

		SuMax	UnMax	SuSum	UnSum
Letter	*Bagging*	74.10 (1%)	73.96 (1%)	73.02 (0%)	**74.94 (2%)**
	*AdaBoost*	73.74 (1%)	73.74 (1%)	72.86 (0%)	**73.76 (1%)**
	*k-NN*	76.44 (11%)	75.72 (4%)	**80.44 (15%)**	75.98 (5%)
Optdigits	*Bagging*	76.60 (0%)	76.60 (0%)	**78.80 (2%)**	76.60 (0%)
	*AdaBoost*	88.90 (15%)	89.30 (17%)	**90.10 (19%)**	88.00 (17%)
	*k-NN*	89.10 (16%)	89.20 (17%)	**90.40 (20%)**	88.00 (20%)
Pendigit	*Bagging*	83.55 (3%)	**84.25 (4%)**	79.90 (0%)	83.55 (3%)
	*AdaBoost*	93.15 (21%)	88.80 (9%)	**94.15 (19%)**	91.85 (13%)
	*k-NN*	92.60 (15%)	91.75 (15%)	**93.65 (18%)**	92.05 (16%)
Statlog	*Bagging*	80.30 (8%)	**84.55 (16%)**	73.70 (1%)	82.20 (10%)
	*AdaBoost*	83.40 (11%)	82.40 (10%)	**85.75 (18%)**	83.80 (12%)
	*k-NN*	85.05 (18%)	84.45 (16%)	**85.80 (19%)**	84.70 (16%)
Vehicle	*Bagging*	**63.00 (2%)**	**63.00 (2%)**	60.00 (1%)	62.00 (1%)
	*AdaBoost*	65.50 (15%)	65.50 (10%)	**67.50 (20%)**	66.00 (15%)
	*k-NN*	64.00 (12%)	63.50 (8%)	**64.50 (6%)**	61.50 (2%)
